# Sirt6 deficiency impairs corneal epithelial wound healing

**DOI:** 10.18632/aging.101513

**Published:** 2018-08-02

**Authors:** Xiaobing Hu, Shuang Zhu, Rong Liu, Jordan D. Miller, Kevin Merkley, Ronald G. Tilton, Hua Liu

**Affiliations:** 1 Wuhan Hanyang Eyegood Ophthalmic Hospital, Wuhan, China; 2 Department of Ophthalmology and Visual Sciences, University of Texas Medical Branch, Galveston, TX 77555, USA; 3 Department of Ophthalmology, Tongji Hospital, Tongji Medical College, Huazhong University of Science and Technology, Wuhan, China; 4 Department of Surgery, Mayo Clinic, Rochester, MN 55905, USA; 5 Department of Internal Medicine, University of Texas Medical Branch, Galveston, TX 77555, USA; 6 Center for Biomedical Engineering, University of Texas Medical Branch, Galveston, TX 77555, USA

**Keywords:** Sirt6, aging, cornea, epithelial wound healing, inflammation

## Abstract

Corneal transparency, dependent on the integrity of epithelial cells, is essential for vision. Corneal epithelial damage is one of the most commonly observed ocular conditions and proper wound healing is necessary for corneal transparency. Sirt6, a histone deacetylase, has been shown to regulate many cellular events including aging and inflammation. However, its specific role in corneal epithelial wound healing remains unknown. Here we demonstrated that Sirt6 was expressed in corneal epithelial cells and its expression decreased with age. In an *in vivo* corneal epithelial wound healing model, Sirt6 deficiency resulted in delayed and incomplete wound healing and was associated excessive inflammation in the corneal stroma and dysfunction of Notch signaling, leading to keratinization of the corneal epithelium and corneal opacity. Aging Sirt6-deficient mice spontaneously developed corneal keratitis with extensive infiltration of inflammatory cells into the cornea. *In vitro* experiments demonstrated that primary corneal epithelial cells with Sirt6 downregulation expressed increased basal levels of inflammatory genes and exhibited hyper-inflammatory reactivity to IL-1β and TNFα treatment. These results provide compelling evidence that Sirt6 is a critical regulator of inflammation in the cornea, and is responsible for corneal epithelial wound healing, thus contributing to the maintenance of epithelial integrity and corneal transparency.

## Introduction

The cornea, an avascular and transparent tissue of the eye, is composed of four main layers: an outer epithelial layer, a middle stromal layer, a Descemet's membrane and an inner layer of endothelial cells [1–3]. A Bowman's layer, which is a thickened acellular collagenous zone, is present in in some species and lies between the epithelium and stroma [3]. The primary function of corneal epithelium is to serve as a protective barrier against physical trauma, pathogens and chemicals, and its structural integrity is essential for the transparency of the cornea and good vision [3,4]. Once epithelium is altered by disease or trauma, it can result in chronic inflammation, keratolysis, deposition of irregular collagen, and further damage to the underlying basement membrane and stroma. Thus, normal epithelial wound healing is essential to maintain the structure and function of the cornea [4]. Wound healing usually accompanies with inflammation that is triggered by injury and the controlled and limited inflammation is necessary for proper wound healing. Nevertheless, excessive inflammation and abnormally persistent recruitment of inflammatory cells are contributing factors for the delayed corneal wound healing, which may lead to corneal opacity and vision loss [5–8].

Sirtuins are evolutionarily conserved NAD+-dependent histone deacetylases and share homologs of yeast Sir2 protein that critically regulates life span of yeast [9]. In mammals, seven different sirtuins (Sirt1–7) have been identified with diverse cellular localizations. Among them, Sirt6 is a chromatin-associated nuclear protein and most closely resembles yeast Sir2 [9,10]. It regulates the acetylation levels of histone H3K9 and H3K56, and has roles in aging, DNA repair, telomere maintenance, gene expression, and regulation of stress response, senescence, and metabolism [11–18]. Both *in vitro* and *in vivo* studies suggest Sirt6 functions to repress inflammation. Overexpression of Sirt6 inhibits NF-κB-regulated inflammatory responses, while the loss of Sirt6 levels/activity was associated with increased NF-κB RelA/p65 activity and amplification of proinflammatory gene expression [12,19,20]. Furthermore, loss of Sirt6 in skin wounds exacerbates diabetes-induced impairment of wound healing [21]. However, the specific role of Sirt6 in corneal homeostasis and function is unknown.

In this report, we demonstrate that Sirt6 is functionally expressed in corneal epithelial cells and required to maintain the integrity of corneal epithelium. After *in vivo* injury, Sirt6 deficiency leads to keratinization of corneal epithelium with excessive inflammation in the corneal stroma, resulting in delayed corneal wound healing. Moreover, Sirt6 expression is decreased in an age-dependent manner, and Sirt6 knockout (KO) mice often spontaneously develop corneal keratitis as they age, characterized by destructive inflammation in the cornea. Deletion of Sirt6 in mouse primary epithelial cells results in the upregulation of inflammatory molecules. Taken together, our findings indicate that Sirt6 is an important regulator of inflammation in the cornea, and is a critical enzyme involved in corneal epithelial wound healing.

## RESULTS

### Sirt6 is expressed in mouse cornea

**Figure 1:**
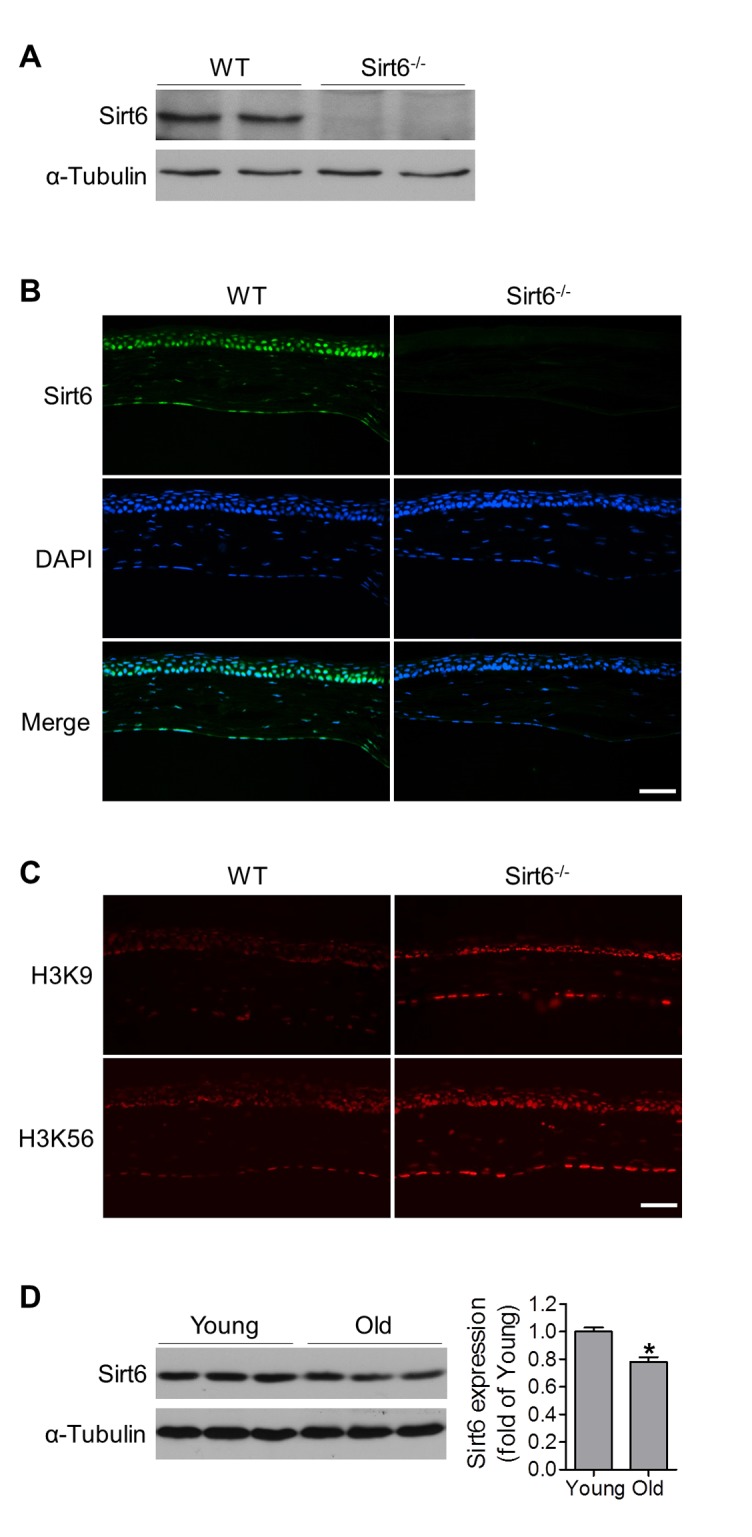
**Functional Sirt6 is expressed in mouse cornea.** (**A**) Protein was extracted from the corneas of WT and Sirt6^-/-^ mice and Sirt6 protein expression was assessed by Western blot analysis. α-Tubulin served as loading control. (**B, C**) Immunostaining of Sirt6 (green), H3K9 and H3K56 (red) in corneal cryosections from 2 month-old WT and Sirt6 KO mice. Blue: DAPI staining for nuclei. Scale bar: 50μm. (**D**) Sirt6 protein expression in young (2 months old) and old (12 months old) mice cornea. Two corneas served as one specimen. *p<0.05, n=4 mice.

In order to study the potential role of Sirt6 in the cornea, we first examined Sirt6 protein expression and its subcellular localization in the cornea by Western blot and immunostaining. We found Sirt6 was expressed in corneal extract from wild type (WT) mice (Fig. 1A) and its immunoreactivity was highest in corneal epithelium although other cells including stromal keratocytes and endothelial cells are also positive for Sirt6 staining (Fig. 1B). The specificity of Sirt6 antibody was confirmed by the absence of signal in the cornea from Sirt6 KO mice. It is known that Sirt6 can deacetylate both histone H3 lysine 9 (H3K9) and lysine 56 (H3K56), two chromatin marks associated with regulation of gene activity [18]. Therefore, we analyzed H3K9 and H3K56 acetylation in corneal sections and found that acetylation of both H3K9 and H3K56 were significantly increased in the cornea from Sirt6 KO mice compared to WT mice (Fig. 1C). Moreover, Sirt6 expression in the cornea was decreased in 12-month old mice (Fig. 1D). Together, our results suggest that Sirt6 is functionally expressed in the mouse cornea and its expression decreases with age.

### Sirt6 deletion impairs corneal epithelial wound healing *in vivo*

**Figure 2:**
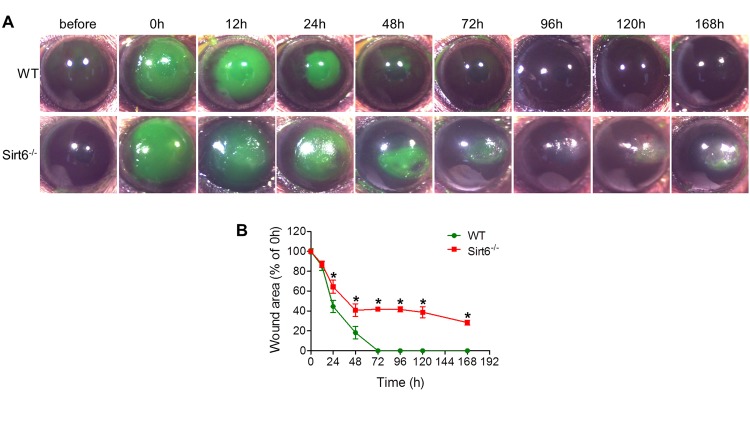
**Corneal wound healing response in WT and Sirt6 KO mice.** 2.0 mm central corneal epithelial debridement wounds were induced in 8-week-old WT and Sirt6 KO mice. (**A**) The corneas were photographed with fluorescein staining to visualize wounds before wounding or at various time points after wound injury. (**B**) The area of each epithelial defect was measured using ImageJ and the wound area was calculated as a percentage of the initial wound area. *p<0.05 compared with WT, n=6-12.

**Figure 3:**
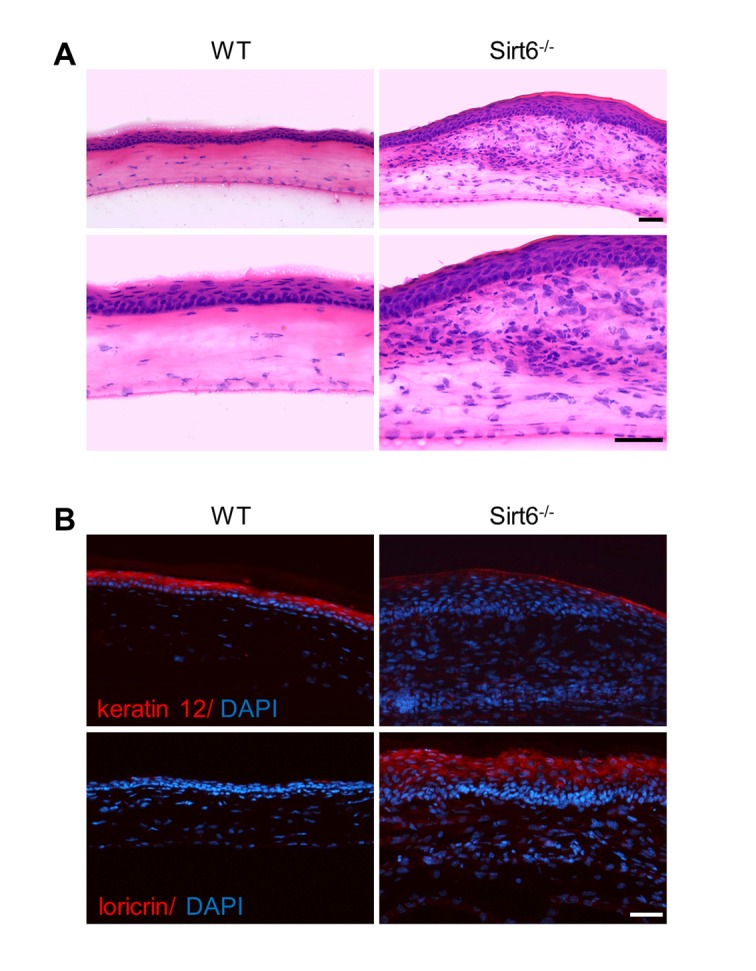
**Pathological changes of the cornea of Sirt6 KO mice after injury.** 2.0 mm central corneal epithelial debridement wounds were induced in 8-week-old WT and Sirt6 KO mice and eyes were collected 3 weeks after injury. (**A**) Histological appearance of corneas with H&E staining at 200X magnification (upper panel) and 400X magnification (lower panel). (**B**) Immunostaining of corneal epithelium marker keratin 12, and the keratinization-related protein loricrin (red) in corneal cryosections from WT and Sirt6 KO mice. Blue: DAPI staining for nuclei. Scale bar: 50 μm.

To characterize the biological roles of Sirt6 in corneal epithelial wound healing, we created a 2-mm corneal epithelial debridement wound mechanically on the corneas of WT and Sirt6 KO mice at 2 months of age. The subsequent corneal wound healing process was recorded kinetically using fluorescein staining. We found that most of the corneal wounds in WT mice closed within 72 hours while the wounds in Sirt6 KO eyes were significantly larger than those in WT eyes at 24, 48 and 72 hours after wounding (Fig. 2). Moreover, at 3 weeks after wounding, WT corneas exhibited normal phenotypes, whereas lack of Sirt6 caused corneal opacity in all KO mice. Histological analysis of H&E stained corneal section revealed that corneal epithelium of Sirt6 KO mice was significantly thickened and keratinized (Fig. 3A). We next examined the corneal epithelium marker keratin 12, and the keratinization-related protein loricrin by immuno-staining. As seen in Fig. 3B, the epithelium of WT corneas specifically expressed keratin 12, which was dramatically decreased in the epithelium of Sirt6 KO cornea. In contrast, loricrin was not expressed in control corneas but highly present in the corneal epithelium of Sirt6 KO. These findings indicate that Sirt6 deficiency results in corneal keratinization after wounding, and suggest that Sirt6 is required for proper wound healing in the cornea.

### Sirt6 deletion upregulates inflammatory response in the cornea after wounding

**Figure 4:**
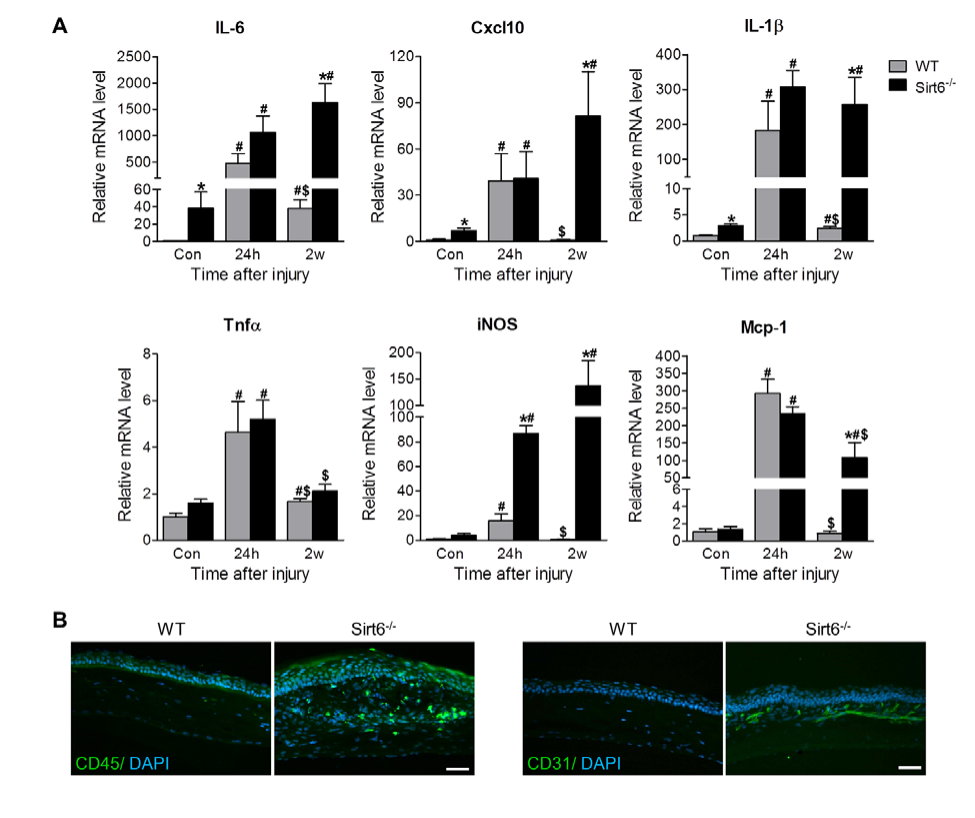
**Sirt6 deletion results in corneal inflammation.** 2.0 mm central corneal epithelial debridement wounds were induced in 8-week-old WT and Sirt6 KO mice and corneas were collected at indicated time pointes after injury. (**A**) Inflammatory genes were assessed by qPCR in WT and Sirt6 KO corneas before (control), 24 hours (24h) and 2 weeks (2w) after wound injury. *p<0.05 compared with WT, #p<0.05 compared with control (con), $p<0.05 compared with 24h. Two corneas were combined and served as one sample at each experimental condition (n=3-4, 6-8 corneas). (**B**) Immunostaining for inflammatory cell marker CD45 and vascular cell marker CD31 (green) in WT and Sirt6 KO corneas 3 weeks after wound injury. Nuclei are counterstained with DAPI (blue). Scale bar: 50 μm.

Acute inflammatory response is an inevitable outcome of tissue injury and plays an important role in normal healing. However, excessive and prolonged inflammation results in delayed healing and increased scar formation [5–8]. Since Sirt6 is involved in inflammation, we hypothesized that prolonged inflammation contributed to impaired corneal wound healing in Sirt6 deficient mice. We assessed corneal mRNA of inflammation-associated genes at different time points, including before wounding, and 24 hours and 2 weeks after wound injury. We found that even before wounding, Sirt6 KO corneas tended to display higher mRNA levels of pro-inflammatory cytokines and chemokines, including IL-6, Cxcl10, IL-1β and iNOS (Fig. 4A). At 24 hours post wound, levels of IL-6, Cxcl10, IL-1β, Tnfα, iNOS and Mcp-1 were significantly increased in corneas of both WT and Sirt6 null mice, but the expression of IL-6 and iNOS was higher in Sirt6 KO corneas than those in control corneas. Two weeks after the wound injury, expression of pro-inflammatory cytokines and chemokines in WT corneas had returned to baseline levels, consistent with the observation that WT cornea exhibited normal morphology (Fig. 3A) and negative immunostaining of CD45 (marker for inflammation) and CD31 (maker for neovascularization) at 3 weeks post wound injury (Fig. 4B). In contrast, the aforementioned genes (except for Tnfα) maintained a sustained high level in Sirt6 KO corneas at 2 weeks after injury (Fig. 4A) and a large number of CD45- and CD31-positive cells were observed in Sirt6 KO corneal stroma at 3 weeks after injury (Fig. 4B).

**Figure 5:**
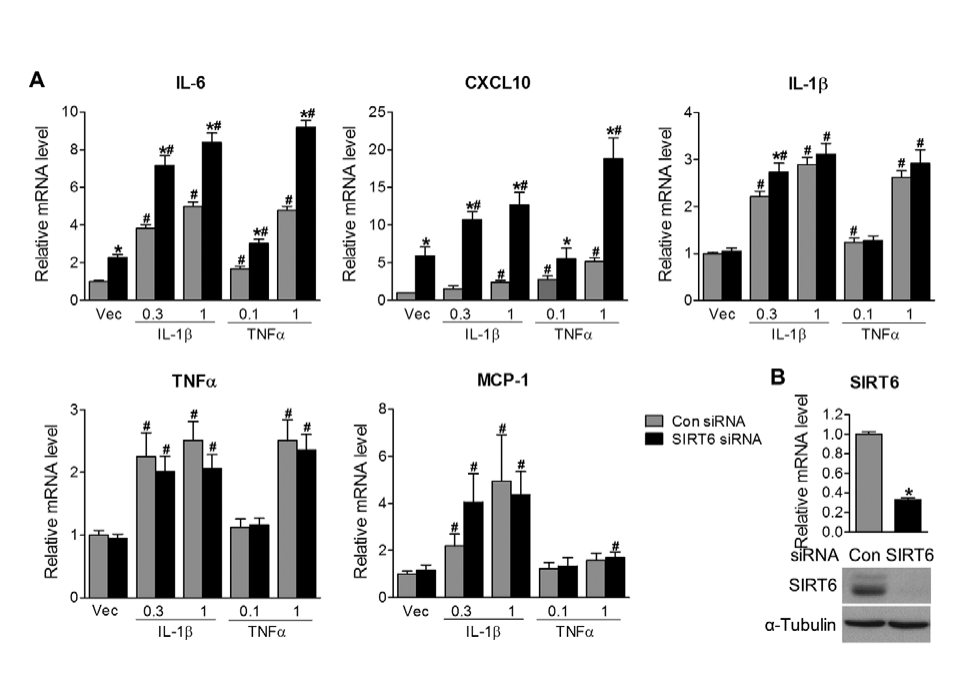
**Sirt6 knockdown induces inflammation *in vitro.*** Human primary corneal epithelial cells (HCEs) were transiently transfected with control siRNA (con) or Sirt6 siRNA. 24 hours after transfection, HCEs were treated with different concentrations of IL-1β (0.3 and 1 ng/ml), TNF-α (0.1 and 1 ng/ml) or vehicle (Vec) for 24 hours. (**A**) qPCR analysis of inflammatory gene expression. *P<0.05 compared to relevant control siRNA; #p<0.05 compared with vehicle, n=3. (**B**) Knockdown of Sirt6 by siRNA in HCEs was confirmed by qPCR (upper panel) and Western blot analysis (lower panel). *p<0.05 compared with control siRNA, n=3 times of independent experiment.

IL-1β and TNFα are two key pro-inflammatory cytokines involved in inflammatory reactions. Their levels are upregulated after corneal injury and overactive IL-1β and TNFα exaggerate corneal inflammation and impair wound healing [22–26]. To determine whether Sirt6 could directly regulate inflammation in corneal epithelial cells, we specifically reduced Sirt6 protein levels using siRNA knockdown in cultured primary human corneal epithelial cells *in vitro* and determined their response to IL-1β and TNFα. We found that cells transfected with Sirt6 siRNA exhibited increased inflammation, as indicated by IL-6 and CXCL10 (Fig. 5A). Moreover, after IL-1β and TNFα treatment, IL-6, CXCL10, IL-1β, TNFα and MCP-1 were upregulated in the cells with control siRNA, but cells with Sirt6 deletion exhibited higher expression of IL-6, CXCL10, IL-1β but not TNFα and MCP-1, suggesting loss of Sirt6 function is associated with increased inflammation. The deletion of Sirt6 was confirmed by qPCR and Western blot (Fig. 5B). Taken together, these findings demonstrated that Sirt6 deficiency induced a pro-inflammatory state in the cornea and prolonged the wound-induced inflammatory responses, resulting in impaired wound healing in the cornea.

### Sirt6 deletion reduces Notch1 signaling

**Figure 6:**
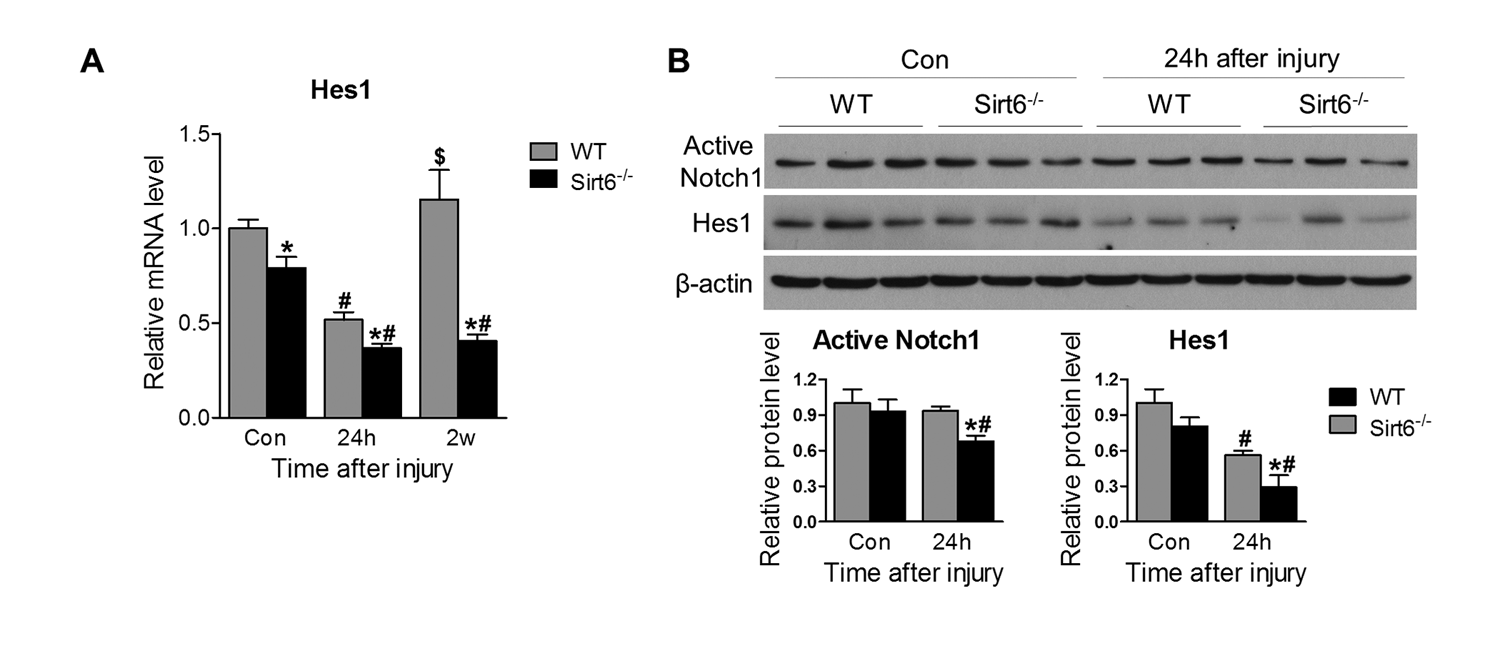
**Sirt6 deletion impairs Notch signaling.** 2.0 mm central corneal epithelial debridement wounds were induced in 8-week-old WT and Sirt6 KO mice and corneas were collected at indicated time points after injury. (**A**) Notch signaling downstream target Hes1 in the corneal was examined by qPCR in WT and Sirt6 KO corneas before injury (control), 24 hours (24h) and 2 weeks (2w) after injury. (**B**) Active Notch1 and Notch signaling downstream target Hes1 in the corneas were examined by Western blot in WT and Sirt6 KO corneas before or 24 hours after injury. *p<0.05 compared with WT, #p<0.05 compared with control (con), $p<0.05 compared with 24h. Two corneas were combined and served as one sample at each experimental condition (n=3-4, 6-8 corneas).

Notch1 signaling is important for corneal homeostasis and maintenance of the non-keratinized corneal cell differentiation and function [1,27]. Therefore, we analyzed Notch1 signaling molecules in the cornea. RT-PCR analysis found that the Notch signaling downstream target gene Hes1 was downregulated in Sirt6 KO mice even prior to injury. While the mRNA expression of Hes1 was decreased in both experimental groups 24 hours after wounding, Hes1 expression returned to baseline levels in WT corneas after 2 weeks whereas it remained markedly suppressed in Sirt6 KO corneas at that time point (Fig. 6A). Consistently, at protein level, Notch signaling as indicated by active form of Notch1 and Hes1 tended to be downregulated in Sirt6 KO mice before injury. After injury, their expression was reduced in WT corneas but Sirt6 deletion exaggerated the reduction (Fig. 6B), suggesting that Notch signaling is dysregulated during wounding by Sirt6 deficiency. These data suggested that Sirt6 may also regulate corneal homeostasis and wound healing through Notch signaling.

### Sirt6 deletion is associated with chronic cornea inflammation and keratitis in aging mice

**Figure 7:**
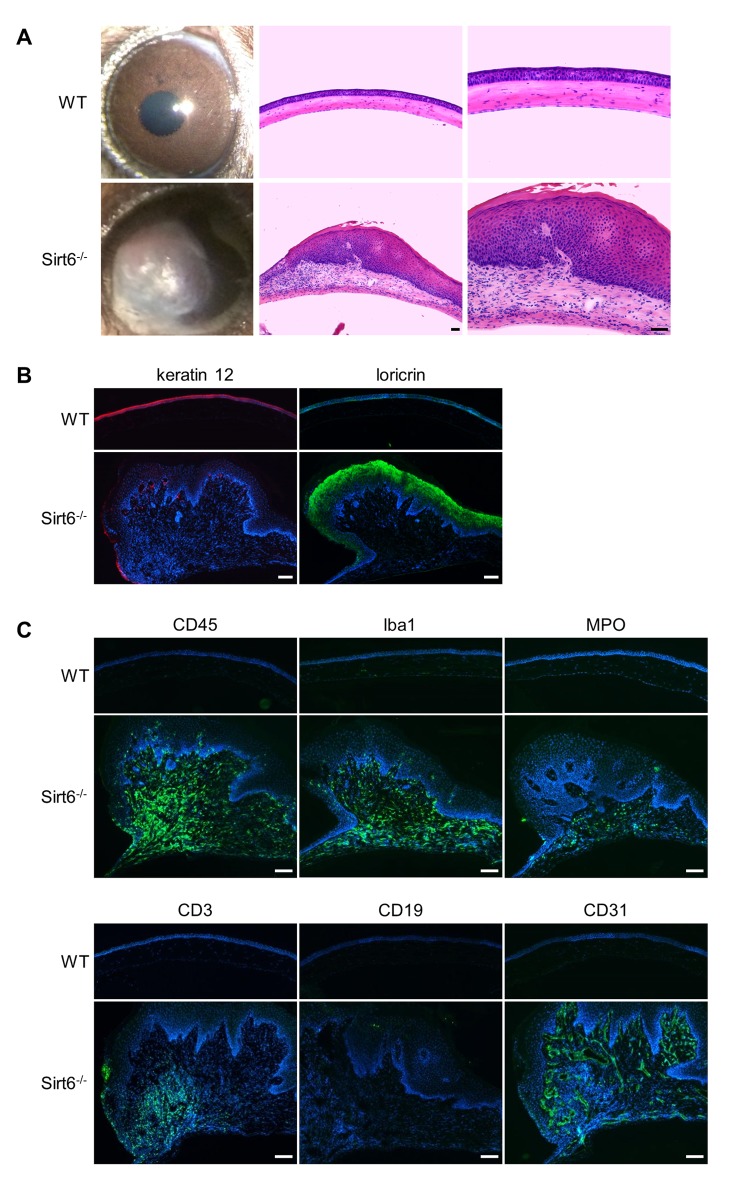
**Sirt6^-/-^ mice develop corneal plaques with infiltration of inflammatory cells and neovascularization as they age.** Eyes were collected from 7-month-old Sirt6 KO mice and their littermate WT mice. (**A**) Images of the corneas and H&E staining of cross-sections of eyes from WT and Sirt6 KO mice (n=6 mice). Scale bar: 50 μm. (**B**) Immunostaining of keratin 12 (red) and loricin (green). Scale bar: 100 μm. (**C**) Immunostaining for hematopoietic cell markers such as CD45 for lymphoid cells (monocytes) (green), Iba1 for activated macrophages, MPO for neutrophils, CD3 for T cells, CD19 for B cells and CD31 for neovascularization in 7 month-old WT and Sirt6 KO corneas. Nuclei are counterstained with DAPI (blue). Scale bar: 100 μm.

In the cornea, structural and functional changes of the corneal epithelium occur naturally with aging, which has a major effect on vision [28–30]. Since Sirt6 expression was decreased in aged cornea (Fig. 1D), we assessed the cornea in WT and Sirt6 KO mice during growth. We found corneal plaques were gradually formed in Sirt6 KO mice at 5-7 months of the age (Fig. 7A) and by 7-10 months of age, about 50% of Sirt6 KO mice developed corneal plaques. Consistent with data from our injury models, abnormal corneal phenotypes in Sirt6 KO mice were manifested by keratinization of the outer corneal layer (Fig. 7B), infiltration of inflammatory cells, including monocytes (CD45^+^), activated macrophages (Iba1^+^), neutrophils (MPO^+^), T cells (CD3^+^), and extensive neovascularization (CD31^+^) (Fig. 7C and Supplementary Figure 1). There was no infiltration of B cells (CD19^+^). These results suggest that Sirt6 deletion enhances aging-induced corneal inflammation and keratinization, and further support our findings that Sirt6 plays an important role in maintaining corneal homeostasis.

## DISCUSSION

Corneal dysfunction due to disease or injury is one of the leading causes of blindness [1]. Proper corneal wound healing is necessary to maintain epithelial integrity and corneal transparency while impaired or delayed wound healing increases the risk of ulceration, adversely impacting vision [1,3,4]. Therefore, it is important to understand how the integrity of the cornea is maintained or reestablished after wound injury.

Sirtuins are a family of NAD-dependent histone deacetylases that contain seven enzymatic members in mammals (Sirt1–7) with diverse cellular localizations and functions [31,32]. Sirt1, Sirt6 and Sirt7 mainly reside in the nucleus and regulate transcription by targeting transcription factors, co-factors or histones; Sirt3, Sirt4 and Sirt5 are predominantly localized within the mitochondria and may regulate mitochondrial energy metabolism; and Sirt2 is primarily found in the cytoplasm where it functions as a tubulin deacetylase. Sirtuins have been shown to be involved in physiological processes and pathologies, such as aging, metabolic diseases, neurodegenerative disorders, cardiovascular disease, kidney disease and cancer [31–33]. Recently, emerging evidence indicates sirtuins are also involved in wound healing, including skin and corneal wound healing. Epidermis-specific deletion of Sirt1 inhibits cutaneous wound healing by regulating cell migration, redox response and inflammation [34] and Sirt6 deficiency delays cutaneous wound healing in diabetic mice [21]. On the other hand, in primary human corneal epithelial cells, overexpression of SIRT1 promotes glucose-attenuated corneal epithelial wound healing through the deacetylation of p53 [35]. Our findings that Sirt6 deficiency results in corneal keratitis and corneal opacity using both an *in vivo* corneal wound healing model and during aging provide further evidence that sirtuins are responsible for the maintenance of corneal homeostasis and function. These findings warrant further exploration of pathophysiological roles of sirtuins and their downstream targets during wound healing.

Corneal wound healing is a complex and tightly controlled process, including cell migration, proliferation, epithelial-mesenchymal cross-talk, and extracellular matrix deposition and remodeling [4]. Although various cytokines or growth factors and neurotransmitters are believed to orchestrate corneal epithelial wound healing, it has been noted that inflammation plays an important role during corneal wound healing [5,6,36]. Generally, proinflammatory mediators, such as Interleukin 1α (IL-1α) and IL-6, are increased at the early stages of wound healing and then decrease through feedback mechanisms. However, prolonged or dysregulated inflammation contributes to delayed corneal wound healing. In the present study that utilized an *in vivo* corneal epithelial wound healing model, we found Sirt6 deficiency resulted in keratinization of the corneal epithelium with excessive inflammation in the corneal stroma, leading to delayed and incomplete wound healing. Moreover, aging Sirt6 KO mice spontaneously developed corneal keratitis with extensive corneal infiltration of inflammatory cells. Subsequent *in vitro* experiments demonstrated that Sirt6 knockdown upregulated inflammation in cultured primary human corneal epithelial cells (HCEs). These results suggest that Sirt6 repression of inflammation is likely one of the responsible pathways in corneal wound healing.

The evolutionarily conserved Notch-signaling pathway plays an important role in development and tissue homeostasis. Other laboratories have reported that Notch1-deficient corneal cells lost their ability to repair injured corneal epithelium [1]. Instead of generating a new cornea after injury, these cells create a hyper-proliferative epidermis-like epithelium, accompanied by progressive inflammation. Our data demonstrated that in Sirt6 deficiency mice, Notch singling is impaired, suggesting Sirt6 may modulate Notch signaling to regulate corneal epithelial homeostasis, contributing to normal structure and repair of wound healing, although the precise mechanisms by which Sirt6 regulates corneal homeostasis and function remain to be elucidated.

In conclusion, the present study demonstrates for the first time that wound healing was significantly delayed in corneas from Sirt6-deficient mice. Moreover, Sirt6 deletion induced a variety of corneal abnormalities, including corneal scarring, vascularization and inflammation, during the normal aging process. Taken together, our data suggest that targeting Sirt6 signaling may be an effective therapeutic strategy to enhance corneal wound healing.

## MATERIALS AND METHODS

### Animals

Animal protocols were approved by the Institutional Animal Care and Use Committee of the University of Texas Medical Branch. All experimental procedures and use of animals were performed in accordance with the Association for Research in Vision and Ophthalmology Statement for the Use of Animals in Ophthalmic and Vision Research. C57BL/6J wild type (WT) mice were purchased from Jackson Laboratory (Bar Harbor, ME), Sirt6 KO and littermate control mice on a C57BL6/129svJ mixed background were generated as described previously [32], and all mice were maintained on a 12:12 light/dark cycle with food and water available *ad libitum*.

### *In vivo* corneal epithelial wounding

Eight-week-old WT and Sirt6 KO mice were anesthetized by intraperitoneal injection of a combination of ketamine hydrochloride (100 mg/kg) and xylazine hydrochloride (10 mg/kg), and proparacaine hydrochloride ophthalmic solution (0.5%) was applied to the eye for local anesthesia before the procedure. Next, a 2-mm puncher was used to demarcate the margins of the wound to avoid damaging the peripheral cornea and limbal areas. To perform the repeated wound, topical fluorescein solution which stains corneal surface when intact epithelial tissue is removed [5] was administered to the corneal surfaces. Then the epithelium within the demarcated area was mechanically removed with a hand-held diamond-tipped glass engraver. The corneas were then photographed with fluorescein staining at different time points after wounding (0, 12, 24, 48, 72, 96, 120 and 168 hours) and the area of each epithelial defect was measured using ImageJ and the wound area was calculated as a percentage of the initial wound area. Finally, mice were euthanized, the eyes were enucleated, fixed in 4% paraformaldehyde (PFA) and embedded in optimal cutting temperature (O.C.T.) compound for corneal frozen sections, or the corneas were excised for protein and mRNA extraction.

### *In vitro* cell culture

Human primary corneal epithelial cells (HCEs) were purchased from American Type Culture Collection (ATCC, Rockville, MD) and cultured in corneal epithelial cell basal media supplemented with corneal epithelial cell growth kit component (ATCC). Cells between passages 2 and 5 were used for all experiments. Cells were maintained at 37°C in a 5% CO_2_ atmosphere, and the culture medium was changed every other day. 6x10^4^ HCEs were plated in 12-well plates, transfected with negative control siRNA (Thermo Fisher Scientific, Waltham, MA) or Sirt6 siRNA (Santa Cruz Biotechnology, Santa Cruz, CA) using Lipofectamine 2000 (Life Technologies, Rockville, MD) according to the manufacturer's protocol. 24 hours after transfection, cells were treated with IL-1β or TNFα for 24 hours. Cells were then collected and processed for quantitative RT-PCR (qPCR) or Western blot.

### Immunostaining

10 μm-thick corneal frozen sections were post-fixed with 4% PFA for 10 minutes, rinsed, and blocked with PowerBlock (Biogenx, San Ramon, CA) for 1 hour. Subsequently, section were incubated with following primary antibodies: anti-Sirt6 and Acetyl-Histone H3 (Lys9) from Cell Signaling Technology (Beverly, MA); anti-Acetyl-Histone H3 (Lys56) from Abcam (Cambridge, MA); anti-MPO from Thermo Fisher scientific; anti-CD3 from eBioscience (San Diego, CA), anti-CD45, CD19 and CD31 from BD Bioscience (San Jose, CA); anti-Iba1 from Wako (Osaka, Japan); anti-keratin 12 from Santa Cruz Biotechnology (Santa Cruz, CA); anti-loricrin from Biolegend (San Diego, CA). After washing, retinal sections were incubated with appropriate Alexa Fluor 488 or 594-labeled secondary antibodies at room temperature for 1 hour. Corneal sections were mounted with Fluoroshield™ with DAPI histology mounting medium (Sigma-Aldrich, St. Louis, MO) and images were taken by fluorescence microscope (Olympus, Waltham, MA).

### Western Blotting

Injured and uninjured corneas were excised from WT and Sirt6 KO eyes at 24 hours after wounding, and were lysed in tissue lysis buffer as described previously [32]. Sample was processed for SDS-polyacrylamide gel electrophoresis. Primary antibodies included anti-Sirt6 (Cell Signaling Technology), activated-Notch1 (Abcam), Hes-1 (Santa Cruz Biotechnology). Mouse monoclonal anti-α-Tubulin or β-actin (Sigma-Aldrich, St. Louis, MO) was used as loading control. Proteins expressions were quantified using ImageJ (NIH, Bethesda, MD). Two corneas served as one specimen at each experimental condition.

### Quantitative real time-PCR (qPCR)

Injured and uninjured corneas were excised from WT and Sirt6 KO eyes at 24 hours or 2 weeks after wounding, and processed for RNA extraction. Two corneas were combined and served as one sample (n=3-4, 6-8 corneas) at each experimental condition. Expression of mRNAs of inflammatory genes and Notch target gene Hes1 in the cornea was assayed by real-time RT-PCR. Primer sequences for mouse and human transcripts were listed in Supplementary Table 1.

### Statistical Analysis

Results are expressed as Mean ± standard error of mean (SEM). Student’s *t*-test was used to compare two groups of animals, whereas ANOVA was used in multiple-group comparisons. Statistical analysis was conducted using GraphPad Prism program (GraphPad Software Inc., La Jolla, CA). *P* < 0.05 was considered significant.

## SUPPLEMENTARY MATERIAL

Supplemental Figure 1

Supplemental Table 1
