# Supplementary material for: Sirt6 deficiency impairs corneal epithelial wound healing
Source: Aging (Albany NY). 2018 Aug 2;10(8):1932–46. doi: 10.18632/aging.101513 (PMC6128418; doi:10.18632/aging.101513)
Supplement: Supplemental Figure 1 [file aging-10-101513-s001.docx]

**
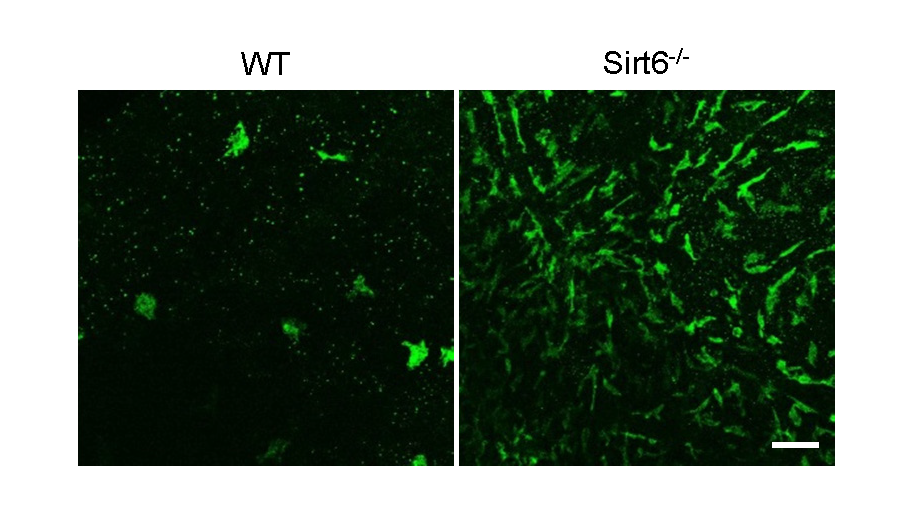
**

**Supplementary Figure 1. Infiltration of inflammatory cells in aged Sirt6-/- cornea.** Eyes were collected from 7-month-old Sirt6 KO mice and WT mice, corneas were excised and subjected to immunostaining
