# Supplementary material for: Sirt6 deficiency impairs corneal epithelial wound healing
Source: Aging (Albany NY). 2018 Aug 2;10(8):1932–46. doi: 10.18632/aging.101513 (PMC6128418; doi:10.18632/aging.101513)
Supplement: Supplemental Table 1 [file aging-10-101513-s002.docx]

**Supplementary Table 1. qPCR primer list.**

| **Name of primer** | **Sequence of forward primer** | **Sequence of reverse primer** |
| --- | --- | --- |
| mouse Hprt | GAAAGACTTGCTCGAGATGTCATG | CACACAGAGGGCCACAATGT |
| mouse Mcp-1 | GGCTCAGCCAGATGCAGTTAA | CCTACTCATTGGGATCATCTTGCT |
| mouse IL-6 | CCACGGCCTTCCCTACTTC | TTGGGAGTGGTATCCTCTGTGA |
| mouse iNOS | GGCAGCCTGTGAGACCTTTG | TGCATTGGAAGTGAAGCGTTT |
| mouse Cxcl10 | CATCCCTGCGAGCCTATCC | CATCTCTGCTCATCATTCTTTTTCA |
| mouse IL-1β | AGTTGACGGACCCCAAAAGA | GGACAGCCCAGGTCAAAGG |
| mouse Tnfα | GGTCCCCAAAGGGATGAGAA | TGAGGGTCTGGGCCATAGAA |
| mouse Hes-1 | CCCCAGCCAGTGTCAACAC | TGTGCTCAGAGGCCGTCTT |
| human HPRT | CCTTGGTCAGGCAGTATAATCCA | GGTCCTTTTCACCAGCAAGCT |
| human IL-1β | GCACGATGCACCTGTACGA | CACCAAGCTTTTTTGCTGTGAGT |
| human IL-6 | AAATTCGGTACATCCTCGACGGCA | AGTGCCTCTTTGCTGCTTTCACAC |
| human CXCL10 | TCCACGTGTTGAGATCATTGC | CGATTCTGGATTCAGACATCTCTTC |
| human TNFα | CCCAGGCAGTCAGATCATCTTC | GCTTGAGGGTTTGCTACAACATG |
| human MCP1 | CTCGCTCAGCCAGATGCAAT | GGACACTTGCTGCTGGTGAT |
| human SIRT6 | TGTGGAAGAATGTGCCAAGTG | ATGGTGCCCACGACTGTGT |
